# Isolation of High Purity Mouse Mesenchymal Stem Cells through Depleting Macrophages Using Liposomal Clodronate

**DOI:** 10.1007/s13770-021-00412-6

**Published:** 2022-01-01

**Authors:** Ju Han Song, Jung-Woo Kim, Mi Nam Lee, Sin-Hye Oh, Xianyu Piao, Zhao Wang, Seung-Hee Kwon, Ok-Su Kim, Jeong-Tae Koh

**Affiliations:** 1 Department of Pharmacology and Dental Therapeutics, School of Dentistry, Chonnam National University, Gwangju, 61186 Republic of Korea; 2 Hard-tissue Biointerface Research Center, School of Dentistry, Chonnam National University, Gwangju, 61186 Republic of Korea; 3 Department of Periodontology, School of Dentistry, Chonnam National University, Gwangju, 61186 Republic of Korea

**Keywords:** Bone marrow, Mesenchymal stem cells, Isolation, Liposomal clodronate, Macrophages, Mouse

## Abstract

*****BACKGROUND***:**

The use of mouse bone marrow mesenchymal stem cells (mBMSCs) represents a promising strategy for performing preclinical studies in the field of cell-based regenerative medicine; however, mBMSCs obtained via conventional isolation methods have two drawbacks, i.e., (i) they are heterogeneous due to frequent macrophage contamination, and (ii) they require long-term culturing for expansion.

*****METHODS***:**

In the present study, we report a novel strategy to generate highly pure mBMSCs using liposomal clodronate. This approach is based on the properties of the two cell populations, i.e., BMSCs (to adhere to the plasticware in culture dishes) and macrophages (to phagocytose liposomes).

*****RESULTS***:**

Liposomal clodronate added during the first passage of whole bone marrow culture was selectively engulfed by macrophages in the heterogeneous cell population, resulting in their effective elimination without affecting the MSCs. This method allowed the generation of numerous high-purity Sca-1^+^CD44^+^F4/80^−^ mBMSCs (> 95%) with just one passaging. Comparative studies with mBMSCs obtained using conventional methods revealed that the mBMSCs obtained in the present study had remarkably improved experimental utilities, as demonstrated by *in vitro* multilineage differentiation and *in vivo* ectopic bone formation assays.

*****CONCLUSION***:**

Our newly developed method, which enables the isolation of mBMSCs using simple and convenient protocol, will aid preclinical studies based on the use of MSCs.

**Supplementary Information:**

The online version contains supplementary material available at 10.1007/s13770-021-00412-6.

## Introduction

Mesenchymal stem cells (MSCs) maintain undifferentiated state through their self-renewal and can further differentiate into various cells types such as osteoblasts, adipocytes, chondrocytes, and neurons under certain conditions [1]. Additionally, owing to their pluripotent properties in combination with their easily accessibility and low immunogenicity, MSCs are considered as potential candidates for developing cell-based therapy for damaged tissues and degenerative diseases [2, 3]. Nevertheless, despite their therapeutic potential, there are stringent restrictions on the accessibility and use of human-derived MSCs for research purposes. To compensate for these issues, MSCs isolated from mice have been actively used as alternative biomaterials in *in vitro* and preclinical studies. Mice are the most commonly used experimental animals because they are economical and easy to manage and manipulate; further, their DNA exhibits high homology to that of humans. Bone marrow (BM) is widely used for isolating mouse MSCs; however, isolation of MSCs from BM is challenging due to the low proportion of MSCs [4].

To date, most of the MSC isolation methods have been based on the property of these cells to attach on the plasticware used for culturing [5, 6]; however, in the case of mouse MSCs, this approach results in contamination with cells of the hematopoietic lineage [7, 8]. To overcome this issue, several culture methods such as frequent medium exchange and low-density culture have been proposed, but these have certain limitations [9–11]. Frequent medium exchange method is inconvenient because the medium needs to be replaced every few hours during the initial culture stage, and low density culture is also associated with both the contamination with hematopoietic lineage cells and the need for a long time (up to months) to obtain sufficiently large numbers of MSCs. In order to solve the issues related to plastic adherence, unique purification methods, such as sorting by using antibodies against specific markers expressed on the cell surface or culture in hypoxic conditions, have been developed by several research groups [12–15]. Although these techniques allow the selection of MSCs with improved functional clonogenicity, they may raise ethical issues as more mice would be sacrificed to obtain a limited yield of MSCs, a very inefficient use of resources. Moreover, these methods require specialized equipment and experienced researcher; hence certain limitations exist when performing them at the individual laboratory unit. Therefore, the establishment of a standardized simple protocol for mBMSC isolation that ensures accessibility and reproducibility is required.

Liposomal clodronate was developed to deplete phagocytes such as macrophages *in vivo* [16]. Once liposomal clodronate is injected into an animal, it is preferentially engulfed by macrophages that recognize liposomes as a foreign antigen, resulting in their depletion. Based on this mechanism of action, liposomal clodronate is actively used in animal studies to assess the role of macrophages in immune or nonimmune responses *in vivo*. Although macrophages account for most of the hematopoietic lineage cells that are isolated along with mouse BMSCs, to our knowledge, an attempt at improving the purity of MSCs by removing macrophages from BMSC cultures using liposome drugs has not been reported *in vitro*. Therefore, in the present study, we describe a simple and effective method for isolating pure MSCs from the mouse BM using liposomal clodronate.

## Materials and methods

### Isolation and expansion of murine BMSCs

In general, 8–10 week old C57BL/6 mice (Damool, Deajeon, Korea) were euthanized by CO_2_ inhalation, and their hind legs were dissected. The tibias and femurs were carefully removed from adherent soft tissues and collected in an ice-cold minimum essential medium (MEM-α; Gibco, Grand Island, NY, USA) supplemented with 10% fetal bovine serum (FBS; Gibco) and 1% penicillin and streptomycin. After transferring the bones into the new complete medium, BM was flushed out from the bone cavity using a 23-gauge needle. The BM suspension was filtered through a 70 µm strainer (SPL, Deajeon, Korea) to remove the bone fragments and debris. The resulting cell suspension originating from one mouse comprised approximately 1 × 10^8^ cells and was adjusted up to 40 ml by adding complete media and seeded into four 10 cm culture dishes (density of 2.5 × 10^6^ cells/ml (5 × 10^5^/cm^2^)). The culture was maintained in a humidified 5% CO_2_ incubator at 37 °C, and was replenished with fresh complete medium every 3 days. By repeating this step, the nonadherent cells can be removed from the culture. On Day 8, when the culture was subconfluent, the attached cells (passage 0; P0) were washed with phosphate-buffered saline (PBS) and then collected by treating with 0.25% trypsin/EDTA (Gibco) for 2 min. For generating liposomal clodronate-treated BMSCs (L-BMSCs), the cells (P0) were seeded at a density of 2 × 10^5^ cells/ml in a medium supplemented with clodrosome (0–0.5 µl/ml; Encapsula Nano Sciences, Brentwood, TN; Cat. No.: CLD-8909), a type of liposomal clodronate. After 24 h of culture, the cells were washed once with PBS and replenished with fresh media, followed by further incubation until confluent. Cell confluence was attained within 5 days of initial culture. Meanwhile, for generating conventional BMSCs (C-BMSCs), cells at P0 were seeded at a density of 1 × 10^5^ cells/ml, replenished with complete media every 3 days, and passaged when they were confluent.

### Flow cytometric analysis

BMSCs were collected by treating with 0.25% trypsin/EDTA and washed once with ice-cold PBS. Thereafter, the cells were probed with fluorescent isothiocyanate (FITC), phycoerythrin (PE), or allophycocyanin (APC)-conjugated monoclonal antibodies, as listed in Supplementary Table S1, in the binding buffer (PBS containing 0.5% FBS and 0.1% sodium azide) at room temperature for 20 min. Next, the cells were washed once with the binding buffer and immediately analyzed using Cytomics FC500 (Beckman Coulter, Miami, FL, USA) with CXP software or Accuri C6 Plus (BD Biosciences, San Jose, CA, USA) with CSampler software. Specific binding of the antibodies was verified by probing the cells with fluorescent dye-conjugated isotype control antibodies (Supplementary Table S1).

### Liposome uptake assay

In C-BMSC culture, the cell-type–specific properties related to liposome ingestion were investigated using Fluoroliposome-DiI (Fluo-DiI; Encapsular NanoSciences, Cat. No.: CLD-8911), a red-fluorescent liposome. Briefly, C-BMSCs were incubated for 16 h in complete medium containing 0.2 µl/ml Fluoro-DiI. Cell groups cultured in the presence or absence of equal concentration of Encapsome (Encapsular Nano Sciences; Cat. No.: CLD-8910), control liposomes, were used as negative controls. Thereafter, the cells were processed for flow cytometric analysis or fluorescent microscopy.

### Fluorescent microscopy

Fluorescence microscopy analysis for cell-type–specific liposome uptake was performed by labeling the cells with Fluo-DiI and probing with antibodies against non-MSC molecules such as CD11b. Briefly, C-BMSCs were incubated with either Encapsome (0.2 µl/ml) or Fluo-DiI (0.2 µl/ml) for 16 h. Thereafter, the cells were fixed with 4% paraformaldehyde (PFA) for 15 min, and probed overnight with Alexa 488-conjugated anti-CD11b monoclonal antibody (1:200). Next, the cells were washed thrice with PBS, and then counterstained with DAPI (5 µg/ml) for 15 min. Images were acquired using Lionheart FX Cell Imager (BioTek, Winooski, VT, USA).

To compare the difference in purity of C-BMSCs and L-BMSCs, both cells were fixed with 4% PFA, and then probed overnight with PE-conjugated anti-CD44 (1:200) and Alexa 488-conjugated anti-CD11b (1:200) monoclonal antibodies. Thereafter, the cells were washed with PBS, counterstained with DAPI, and observed under Lionheart FX Cell Imager.

### Differentiation induction

To induce osteogenic differentiation, BMSCs were seeded at a density of 1 × 10^4^ cells/cm^2^ in complete medium. On the subsequent day, the cells were exposed to the osteogenic induction medium containing 50 µg/ml ascorbic acid (Sigma, St. Louis, MO, USA), 10 mM β-glycerol phosphate (Sigma), and 50 ng/ml human bone morphogenetic protein 2 (BMP-2; Cowellmedi, Pusan, Korea) in MEMα, and the induction medium was replaced every 3 days. The control cells were cultured in normal growth media. On Day 15 after the differentiation induction, the cells were fixed with 4% PFA and stained with Alizarin Red S (Sigma) to evaluate the culture mineralization.

To induce adipogenic differentiation, 2 × 10^4^ cells/cm^2^ BMSCs were seeded in complete medium and cultured overnight. From the subsequent day, the adipogenic induction medium, comprising 1 µM dexamethasone (Sigma), 2 µM rosiglitazone (Sigma), and 10 µg/ml insulin (Sigma) in MEMα, was used to replenish the cells every 3 days. After 9 days of induction of differentiation, the cells were fixed with 4% PFA, stained with Oil Red O (Sigma) and observed by light microscopy. The Oil Red O-stained lipid droplets inside cells were further dissolved using absolute isopropanol and its absorbance was measured at 510 nm. Adipogenic differentiation was also determined using Bodipy 493/503 fluorescent dye (Thermo Fisher Scientific, Waltham, MA, USA) staining, according to the manufacturer’s instruction.

### Ectopic bone formation study

To estimate the ectopic bone formation capacity, 1 × 10^6^ of either C-BMSCs or L-BMSCs were mixed with 100 µl Matrigel (Corning, Bedford, MA, USA) containing 1 µg hBMP-2 on ice. Each of the cell mixtures was subcutaneously implanted in two different hind flanks of 8-wk-old C57BL/6 female mice. The Matrigel mixture without BMSCs was injected as a control. Two weeks after injection, the mice were anesthetized and analyzed using an *in vivo* micro-computed tomography (µ-CT) system (Quantum FX MicroCT, PerkinElmer, Waltham, MA, USA) to obtain 3-dimensional (3D) images of the newly formed bone. To further analyze detailed characteristics of the bone, ectopic tissues from the mice were isolated and fixed in 4% PFA and scanned using an ex vivo Skyscan1172 μ-CT system (Skyscan, Aartselaar, Belgium) at pre-optimized setting (50 kV, 0.2 mA, and 0.5 mm aluminum attenuation filter) [17]. Scan images were then analyzed using imaging software programs, such as NRecon (Skyscan) and Mimics (Materialise, Leuven, Belgium). For histological analysis, the tissue samples after µ-CT analysis were decalcified in a decalcifying agent (Calci-Clear Rapid, National Diagnostics, Atlanta, GA, USA), embedded in paraffin, sectioned at 5 µm thickness, and then stained with hematoxylin and eosin (H&E).

### RNA extraction and quantitative RT-PCR

Total RNA from cells was extracted using TRIzol reagent (Ambion, Carlsbad, CA, USA). Two micrograms of total RNA were reverse-transcribed into cDNA using M-MLV reverse transcriptase (Promega, Madison, WI, USA). The expression profiles of cell-type–specific markers in the cDNA samples were accessed using QuantStudio cycler (Applied Biosystem, Valencia, CA, USA) with specific primers (Supplementary Table S2) and Power SYBR Green PCR Master Mix (Applied Biosystem). Data were presented as the relative expression of the ΔΔCt value acquired by QuantStudio Design & Analysis software (Applied Biosystem). The 18S rRNA was used as an internal control.

### Statistical analysis

Data were obtained from at least two-independent experiments. Significance of the difference between each group was determined using a paired Student’s *t*-test. A *p-*value less than 0.05 was considered as significant.

## Results

### mBMSCs isolated by a conventional method are contaminated from macrophage-like cells

**Fig. 1:**
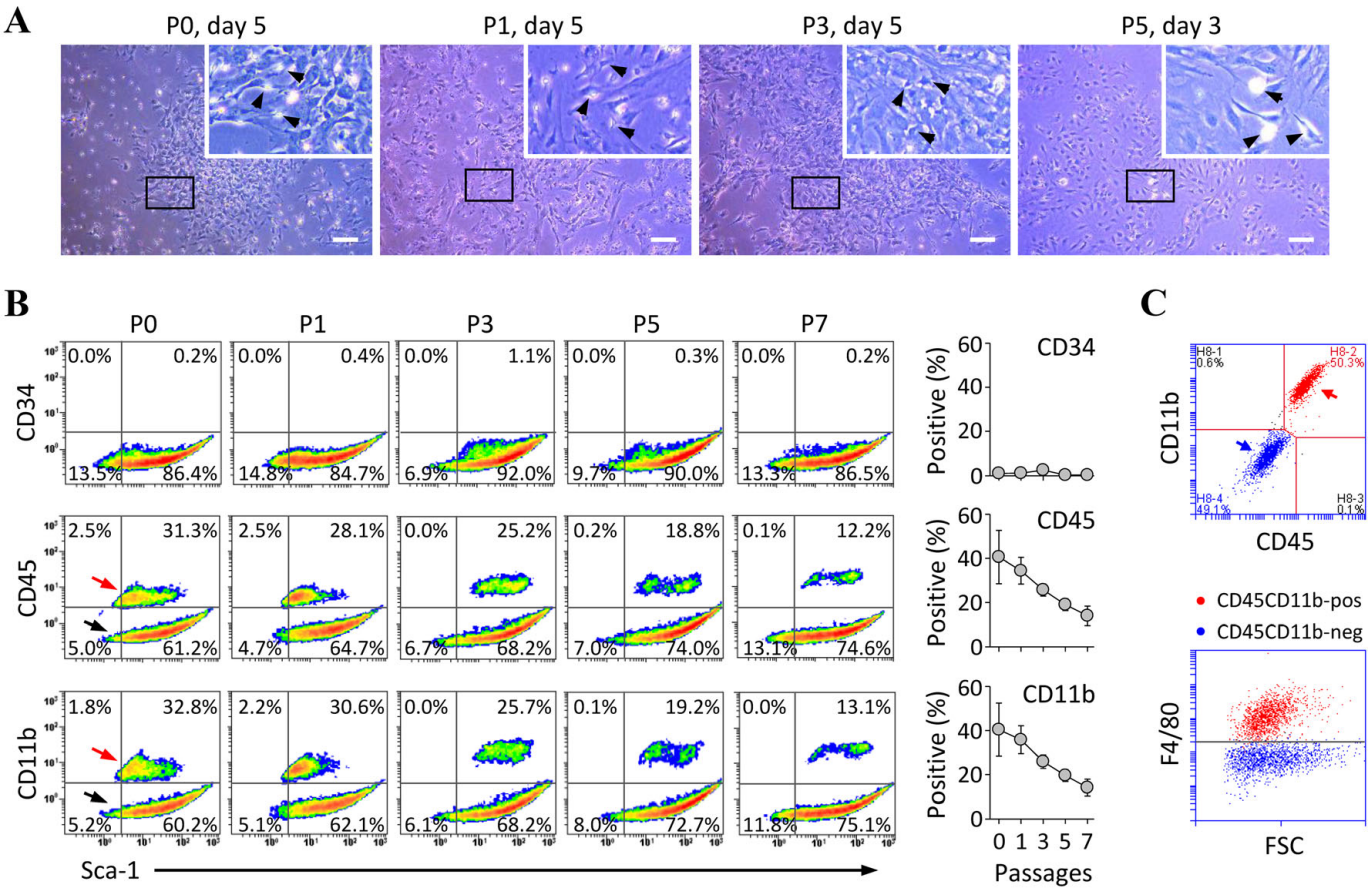
mBMSCs isolated using a conventional method are contaminated with macrophage-like cells. **A** Representative images of C-BMSC culture at the indicated passage. Arrowheads indicate macrophage-like cells. Original magnification, 5 × . Scale bar, 200 µm. **B** Flow cytometric analysis of C-BMSCs at the indicated passage. Cells were collected when they were confluent and double-stained with fluorescent antibodies against Sca-1, CD34 (hematopoietic precursor-specific marker), CD45 and CD11b (myeloid-specific markers). The data show a representative density plots (left) and the mean ± S.D. (right) from three-independent experiments. Red and black arrows indicate macrophage-like cell and MSC population, respectively. **C** C-BMSCs at passage 1 were subjected to flow cytometry after staining with fluorescent antibodies against CD45, CD11b, and F4/80 (macrophage-specific marker). The blue and red arrows indicate F4/80-negative cells (i.e., MSCs) and F4/80-positive cells (i.e., macrophage-like cells), respectively. FSC, forward scatter

To clarify whether MSC cultures generated using the conventional method are contaminated from non-MSCs, we evaluated cellular heterogeneity in a culture of conventional BMSCs (C-BMSCs) based on the concept of plastic adhesion. Microscopy revealed that cells attached on the surface of the plasticware used for culture were heterogeneous although they were passaged thrice (Fig. 1A). To further define the cell type, antigens expressed on the cell surface were analyzed using flow cytometry. We confirmed that numerous cells were positive for the presence of hematopoietic lineage markers such as CD45 or CD11b, although the positive cell population decreased upon repeated subculturing (Fig. 1B). The results obtained after triple-immunostaining with CD45, CD11b, and F4/80 (macrophage-specific marker) clearly indicated that the non-MSCs are macrophages among myeloid lineage cells (Fig. 1C). These data indicate that the commonly used method needs to be improved.

### Macrophage contaminants in mBMSC culture exhibit an active antigen uptake property

**Fig. 2:**
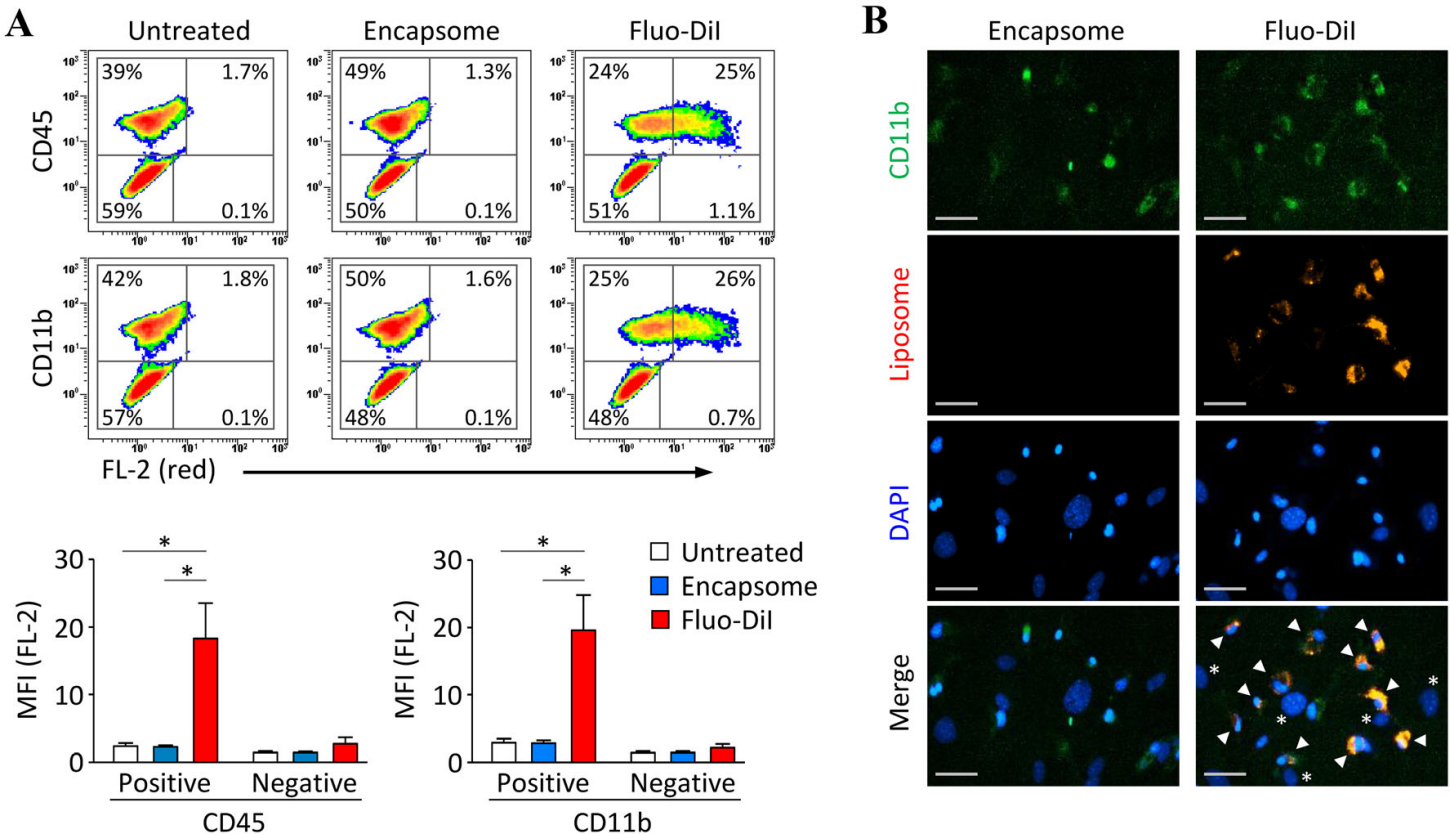
Macrophage-like cells preferentially ingest liposomes in the C-BMSC culture. C-BMSCs at passage 1 were treated with either non-fluorescent control liposome (Encapsome, 0.2 µl/ml) or red-fluorescent liposome (Fluo-DiI, 0.2 µl/ml) for 16 h. **A** Cell suspensions were stained with FITC-conjugated anti-CD45 or CD11b antibodies and immediately subjected to flow cytometry. The representative density plots (upper panel) for cell-type specific liposome ingestion are depicted, and the mean fluorescent intensity (MFI; lower panel) is expressed as the mean ± S.D., from three-independent experiments. FL-2, fluorescent light-2. **B** The cells were stained with Alexa 488-conjugated anti-CD11b antibody and DAPI and observed by fluorescent microscopy. Arrow heads and asterisks indicate macrophages and MSCs, respectively. Original magnification, 10 × . Scale bar, 50 µm

As macrophages are representative phagocytic cells, we tried to utilize liposomal clodronate to remove the contaminant macrophages from the mBMSC culture. Initially, we investigated the cell-type–specific liposome intake in C-BMSC culture comprising BMSCs and macrophages in the presence of fluorescent-labeled liposomes (Fluo-DiI). As demonstrated via flow cytometry, the cells positive for the expression of CD45 or CD11b aggressively phagocytosed liposomes, whereas the cells negative for the expression of these molecules did not (Fig. 2A). Concordantly, immunocytochemistry revealed that liposomes were selectively ingested by CD11b-positive cells in the C-BMSC culture (Fig. 2B). These results strongly support our hypothesis that liposomal clodronate can be used for eliminating macrophages during the isolation and expansion of mBMSCs.

### Liposomal clodronate selectively eliminates macrophages in mBMSC culture

**Fig. 3:**
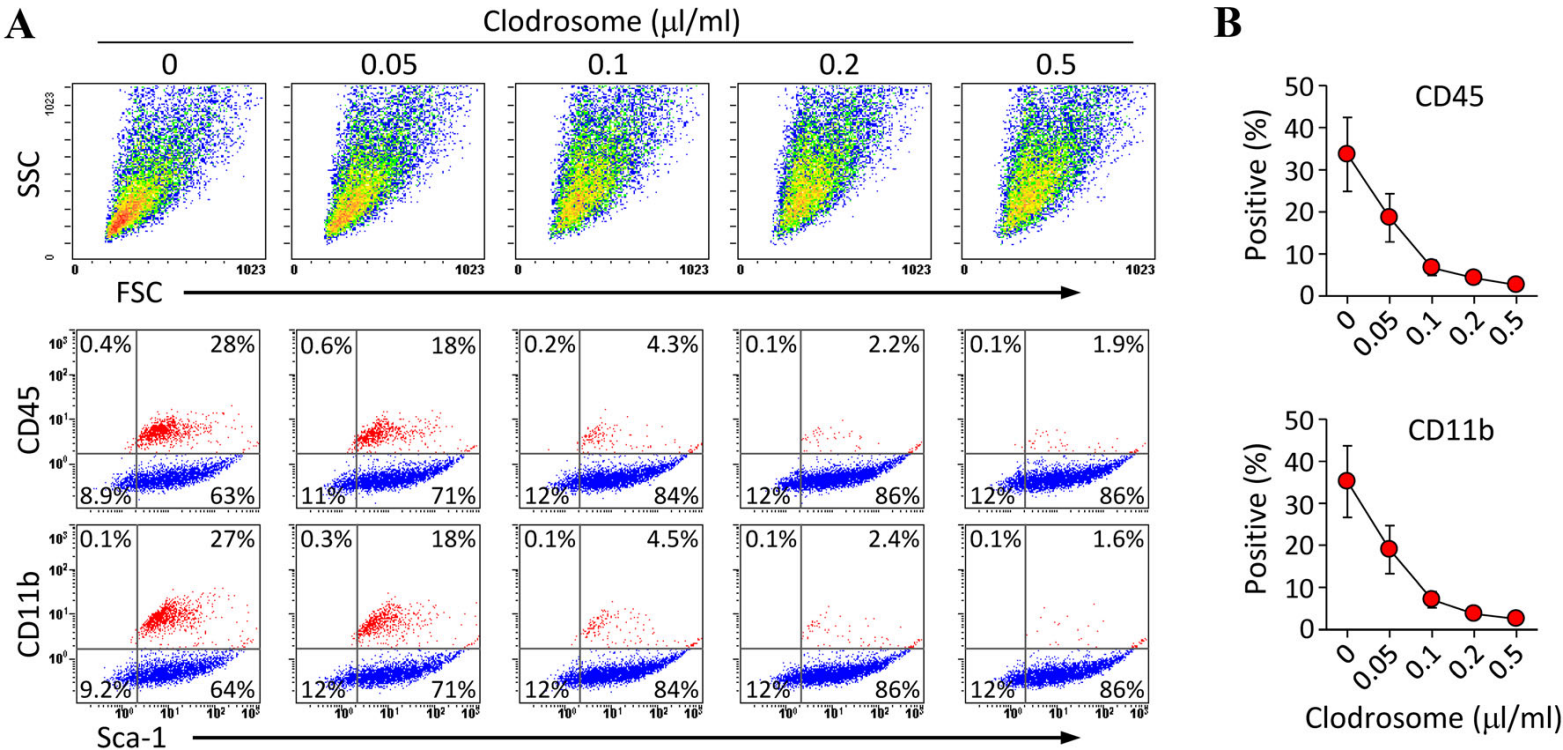
Liposomal clodronate dose-dependently depletes macrophages in C-BMSC cultures. C-BMSCs at passage 0 were trypsinized and reseeded in a medium supplemented with the indicated concentration of clodrosome. After 24 h of initial incubation, the cells were harvested, stained with fluorescent antibodies against Sca-1, CD45, and CD11b, and then analyzed using flow cytometry. **A** The representative plots are depicted. FSC, forward scatter; SSC, side scatter. **B** The graphs indicate the percentage of CD45- or CD11b-positive cells and are expressed as the mean ± S.D. Data were obtained from three-independent experiments. Red spots indicate the population of macrophage-like cells

Considering our *in vitro* observations that macrophages selectively ingest liposomes, we further tried to evaluate the effect of liposomal clodronate (clodrosomes) with respect to removing macrophages from the conventional BMSC culture. Clodrosomes were used once in the step of first passaging of the C-BMSCs (P0). The culture of C-BMSCs in the medium supplemented with clodrosome resulted in a concentration-dependent decrease in the number of CD45- or CD11b-positive cells, as evidenced by flow cytometry (Fig. 3). Based on the results of this experiment, we selected 0.2 µl/ml as a suboptimal clodrosome concentration and used it for future studies.

**Fig. 4:**
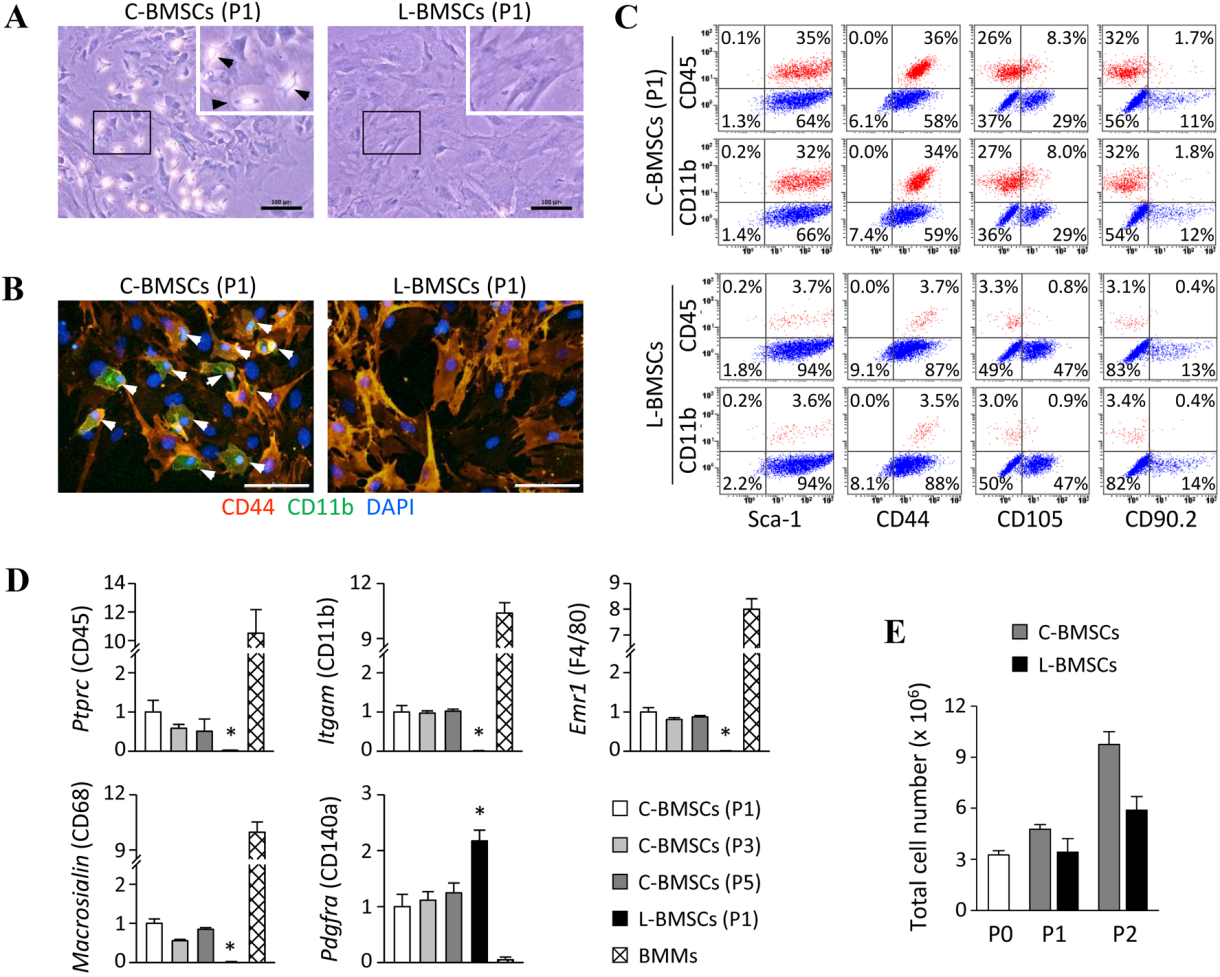
Morphological and immunophenotypical characteristics of L-BMSCs. L-BMSCs were generated by treating C-BMSCs with clodrosome (0.2 µl/ml) for 24 h. **A** Light microscopy-based images of the cell cultures. Arrowheads indicate macrophage-like cells. Original magnification, 10 × . Scale bar, 100 µm. **B** Representative fluorescent microscopy-based images of C-BMSCs and L-BMSCs probed with antibodies against CD44 (orange) and CD11b (green). Nuclei were labeled with DAPI (blue). Arrowheads indicate macrophage-like cells. Original magnification, 20 × . Scale bar, 100 µm. **C** Cell surface markers were evaluated by flow cytometry. Red spots indicate the population of macrophage-like cells. Data are representative of three-independent experiments with a similar result. **D** Real-time PCR for checking the expression of macrophage (*Ptprc*, *Itgam*, *Emr1*, and *CD68*) or MSC (*Pdgfra*) marker genes was performed in the indicated cells. The values are expressed as the mean ± S.D (*n* = 3), relative to those in C-BMSCs (P1). BMMs, bone marrow-derived macrophages. *p < 0.01 versus C-BMSCs (P1). **E** Total cell number were collected at the indicated passages of both cell cultures. The data are expressed as the mean ± S.D (*n* = 2)

Moreover, we assessed the improvement in BMSC purity following the treatment with clodrosome. As illustrated in Fig. 4A, liposomal clodronate-treated BMSCs (L-BMSCs) were markedly homogenous compared to C-BMSCs. Furthermore, unlike C-BMSCs that included a combination of cells positive for the expression of CD11b and CD44, L-BMSCs were markedly homogenous comprising cells positive for the expression of CD44, as evidenced by fluorescence microscopy (Fig. 4B). Next, the distribution of the cell populations isolated using the two distinct methods was quantified via flow cytometry. We confirmed that > 95% of the cells in the L-BMSC culture were MSCs, which were negative for the expression of CD45 and CD11b; whereas, approximately less than two-thirds of the cells in the C-BMSC culture were MSCs, which are negative for the expression of both markers (Fig. 4C). The expression of general MSC markers, such as Sca-1, CD44, CD105, and CD90.2, was in agreement with that observed in previous studies [18, 19]. Macrophage elimination from BMSC culture was also verified by checking the expression of either myeloid cell or MSC marker genes via quantitative PCR. The expression of myeloid lineage cell markers, such as *Ptprc* (CD45), *Itgam* (CD11b), *Adgre1* (F4/80), and *Macrosialin* (CD68), was detected in all C-BMSC cultures obtained at passages 1, 3, and 5 but not in L-BMSC (P1) culture (Fig. 4D). In contrast, the expression of *Pdgfra*, a MSC marker [20], was relatively higher in L-BMSC cultures rather than that in C-BMSC cultures. To verify that the clodrosome selectively removes macrophages, we further measured the total number of cells obtained from each BMSC culture cultivated with or without clodrosome treatment. We confirmed that the number of clodrosome-treated L-BMSCs was reduced by the proportion (approximately 40%) of macrophages identified in the FACS analysis, compared to that of C-BMSCs (Fig. 4E). These results demonstrate that the newly developed method can generate highly pure MSCs from which the non-MSCs have been effectively removed.

### L-BMSCs exhibit an enhanced differentiation potential *in vitro*

**Fig. 5:**
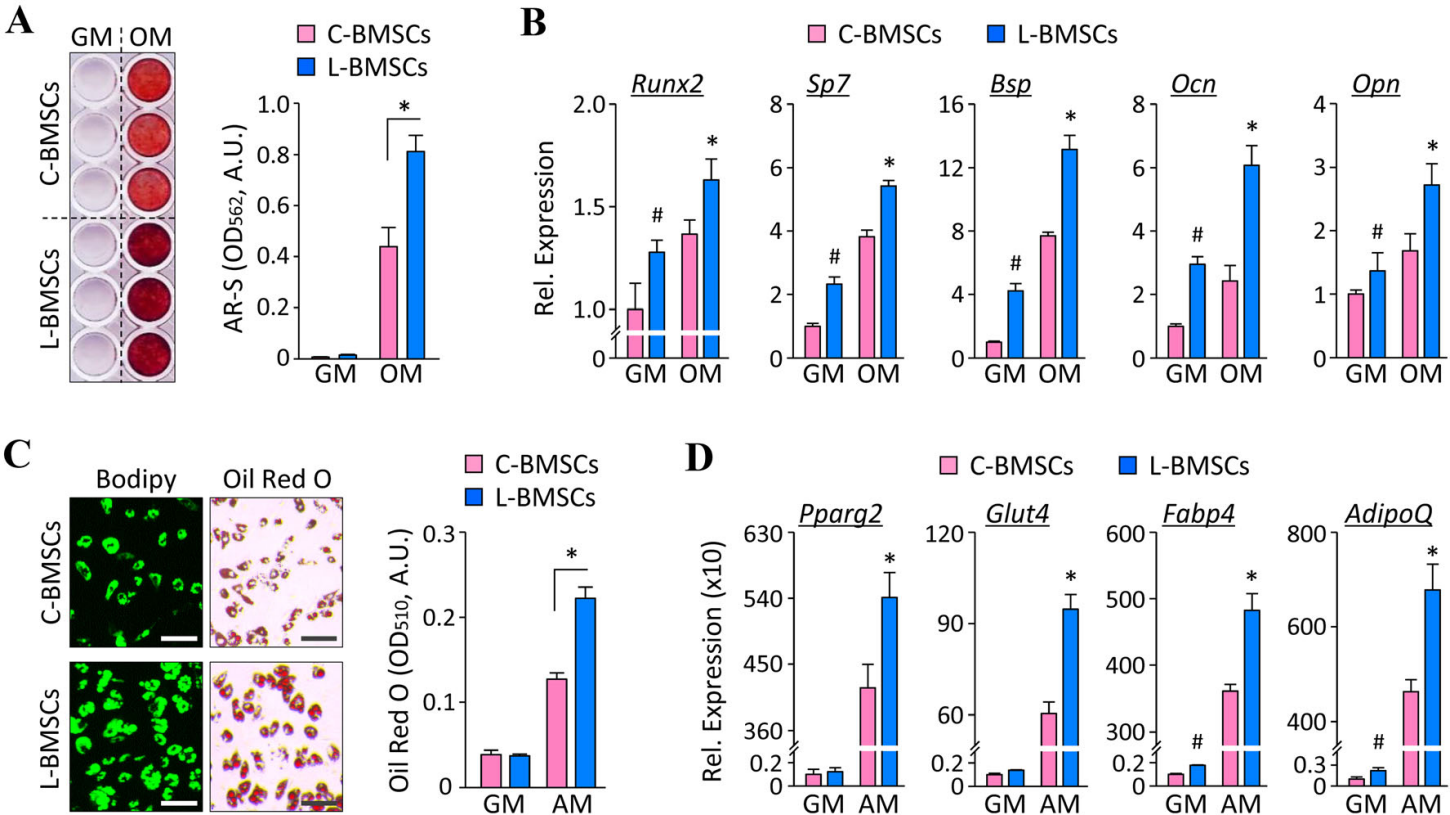
Lineage differentiation potential of L-BMSCs. C-BMSCs (P1) and L-BMSCs were cultured in osteogenic (OM) or adipogenic (AM) differentiation induction medium. Cells cultured in normal growth medium (GM) served as control. **A** Osteogenic differentiation was evaluated on Day 15 by Alizarin Red S staining (left) and by measuring its absorbance (right). **B** On Day 4 after the induction of differentiation, the expression of osteogenic differentiation markers was determined by real-time PCR. Data are expressed as the mean ± S.D. (*n* = 3). **C** Adipogenic differentiation was evaluated on Day 8 by Bodipy 493/503 and Oil Red O staining (left). Graph represents the contents of Oil Red O dye (right). Scale bar, 100 µm. **D** On Day 6, after the induction of differentiation, the expression of adipogenic differentiation markers was determined by real-time PCR. Results are expressed as the mean ± S.D. (*n* = 3). ^#^*p* < 0.05 versus C-BMSCs cultured in GM and *p < 0.05 versus C-BMSCs cultured in differentiation induction medium

To compare the functional potency between C-BMSCs and L-BMSCs, we induced the differentiation of these cells into either osteoblasts or adipocytes. When the cells were differentiated into osteoblasts, L-BMSCs exhibited abundant extracellular calcium accumulation compared to that observed in C-BMSCs, as evidenced by Alizarin Red S staining (Fig. 5A). Moreover, in accordance with the results of cell mineralization, the mRNA expression of osteogenic differentiation markers, including *Runx2*, *Sp7*, *Bsp*, *Ocn*, and *Opn* was relatively higher in L-BMSCs compared to that in C-BMSCs (Fig. 5B). Furthermore, during adipogenic differentiation, a higher number of intracellular lipid droplets was observed in L-BMSCs than that in C-BMSCs, as evidenced upon staining with Bodipy 493/503 fluorescent dye and Oil Red O dye (Fig. 5C). As expected, the expression of adipogenic differentiation-specific markers, such as *Pparg2*, *Glut4*, *Fabp4*, and *AdipoQ* was remarkably higher in L-BMSCs than that in C-BMSCs (Fig. 5D).

### L-BMSCs exhibit enhanced ability for ectopic bone formation *in vivo*

**Fig. 6:**
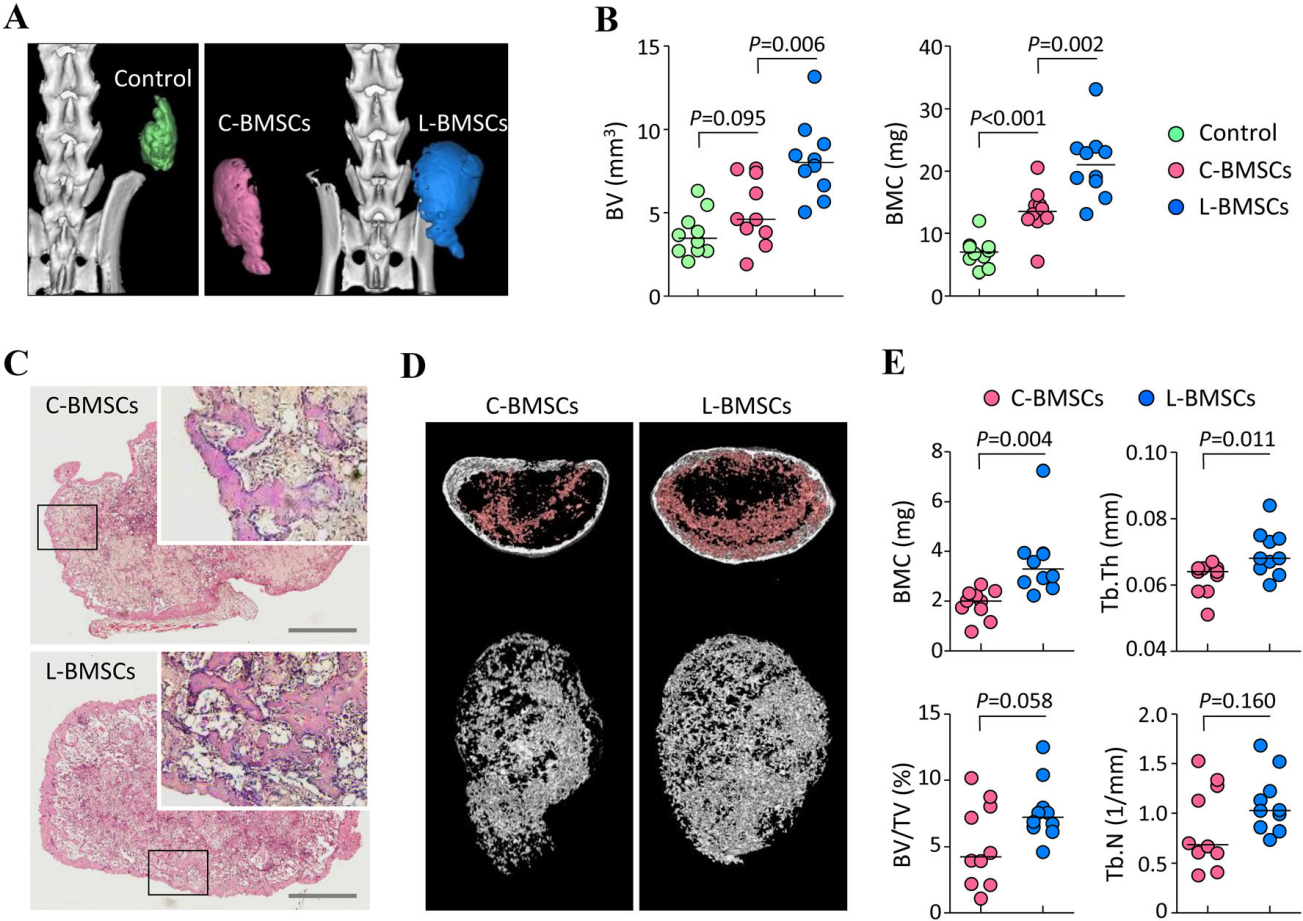
Ectopic bone formation capacity of L-BMSCs. Ectopic bone formation was investigated in mice 14 days after the implantation of Matrigel mixed with or without BMSCs. **A** Representative 3-dimensional structure of the ectopic bones obtained using *in vivo* µ-CT system. **B** Ectopic bone samples were isolated from mice and their whole masses were measured by µ-CT analysis. BV, bone volume; BMC, bone mineral content (*n* = 10). **C** Representative images for H&E staining in ectopic bone slides. Scale bar, 1 mm. **D**,**E** Three-dimensional structure of the trabecular bones (**D**) and their quantitative parameters (**E**) in the ectopic samples were assessed via additional µ-CT analyses (*n* = 10)

To investigate the ectopic bone formation potential of L-BMSCs and C-BMSCs, we injected BMP-2-containing Matrigel mixed with or without the cells into mice. Two weeks after injection, the mice were analyzed using the *in vivo* µ-CT system to monitor the newly formed bones, and the 3D images indicated that ectopic bones were present in all experimental groups (Fig. 6A). To characterize the ectopic bone tissues, we isolated them from the mice and further analyzed them using a µ-CT scanner. As expected, the bone tissues formed by the Matrigel mixture containing either C-BMSCs or L-BMSCs revealed increased bone volume (BV) and bone mineral contents (BMC), compared to those formed by the Matrigel mixture without BMSCs (Fig. 6B). Importantly, the ectopic tissues formed by the L-BMSC-containing mixture exhibited remarkably higher BV and BMC compared to those formed by the C-BMSC-containing mixture (Fig. 6B). To further confirm that these cells differently affected trabecular bone formation, the ectopic bones were sectioned and stained with H&E. We found that the inside matrix of ectopic bones derived from the L-BMSC mixture had thicker trabecular bones than that derived from the C-BMSC mixture (Fig. 6C). Based on this observation, we further performed a separate quantitative analysis restricted to the trabecular bone area of ectopic tissues, wherein, we confirmed that the injection of L-BMSCs rather than that of C-BMSCs markedly affected the improvement in BMC and trabecular bone thickness (Tb.Th) during trabecular bone formation (Fig. 6D, E). Moreover, bone parameters, such as bone volume per tissue volume ratio (BV/TV) and trabecular bone number (Tb.N), tended to increase when L-BMSCs were used, although the results were not significant (Fig. 6E). These results suggested that our separation method can generate mBMSCs that have functionality as well as utility in *in vitro* and *in vivo* studies.

## Discussion

**Fig. 7:**
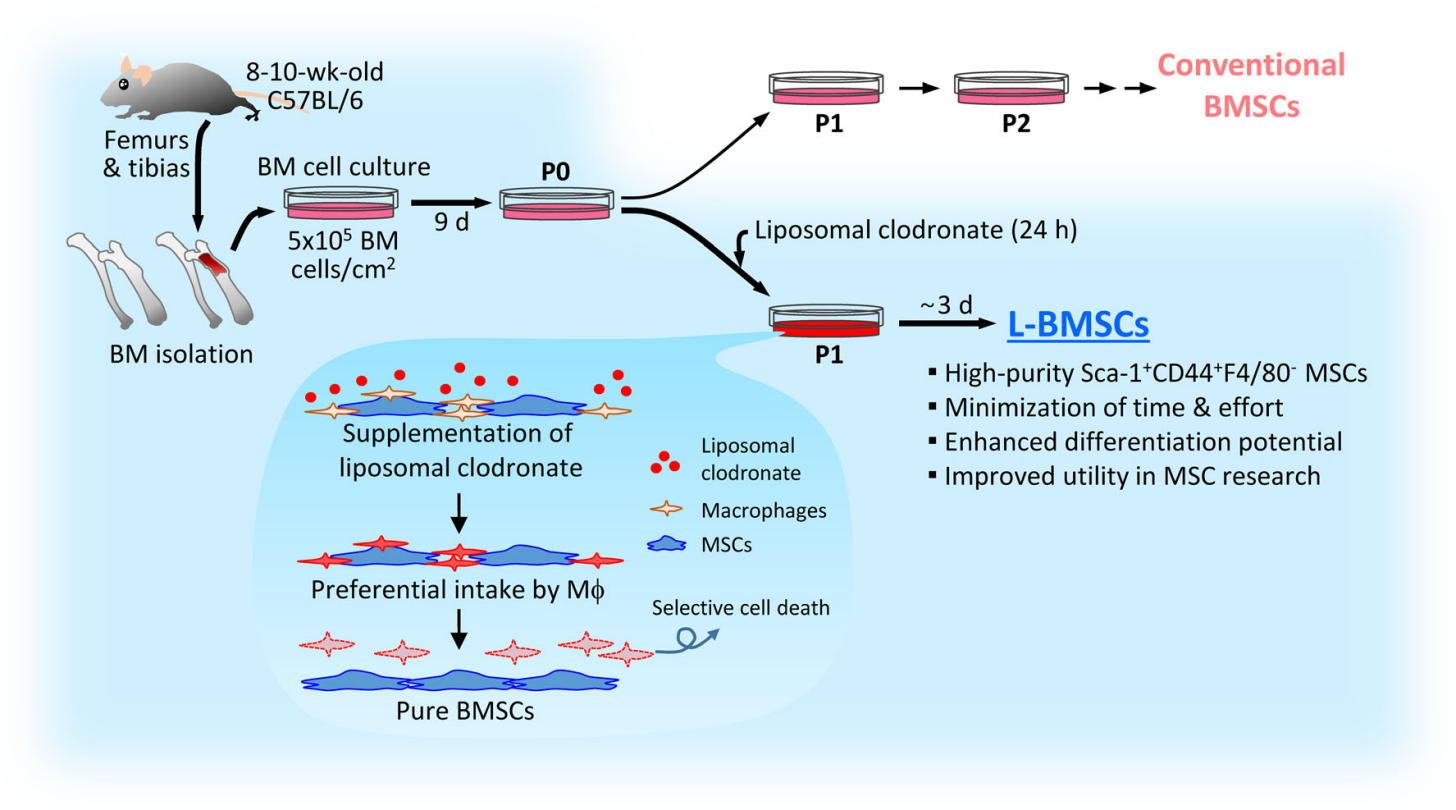
A novel strategy for pure mBMSC isolation by using liposomal clodronate

Although mBMSCs have immense value in various experimental applications, the establishment of an efficient and standardized method for isolating and expanding mBMSCs is still being investigated. In particular, the most common method of mBMSC separation that relies on the property of MSCs to adhere to plastics used in cell culture has a disadvantage, in that macrophage contamination and long-term culture cannot be avoided. In the present study, we designed a unique and simple method to selectively eliminate macrophage contamination during isolation and expansion of mBMSCs. Furthermore, we proved that this approach enables the acquisition of a sufficient amount of high-purity mBMSCs with a short culture duration without supplementation of growth factor, and eventually, mBMSCs generated using this method can be employed in various *in vitro* and *in vivo* functional assays (Fig. 7).

Moreover, we tried to solve the problems associated with the conventional mBMSC separation method by focusing on reducing the contamination with non-MSCs. Initially, we experimentally identified that most of these cells exhibit macrophage-like properties (Fig. 1C). Next, we confirmed that repeated passaging by controlled trypsinization of the BMSC culture, starting from the BM cells gradually reduces the number of macrophages and increases the percentage of MSCs (Fig. 1). Nevertheless, this method required at least seven subcultures to obtain high-purity MSC cultures that contained less than 10% macrophages, implying that the biological properties of MSCs may be altered upon long-term culture [21, 22]. Furthermore, because the functional properties of MSCs are altered upon interaction with macrophages [23, 24], macrophage contamination may hinder the attainment of reproducible and reliable results in studies utilizing MSCs.

Therefore, we tried to find an easy approach to effectively remove macrophages. We presumed that liposomal clodronate could be used as a tool to eliminate macrophage contamination from *in vitro* mBMSC cultures. Therefore, although the mechanism of action of liposomal clodronate was unlikely to cause problems *in vitro*, we carefully examined the cell-type specific action of liposomal drugs in our experimental conditions. As illustrated in Fig. 2, the preliminary experiments using fluorescent liposome strongly supported our hypothesis that liposomes are preferentially internalized by macrophages rather than by MSCs in *in vitro* BMSC cultures containing a heterogeneous cell population. Based on this finding, we confirmed that treatment with clodrosome—a commercially available liposomal clodronate—at an appropriate concentration for 24 h—after the first passage of cultured whole BM cells—removes macrophages from BMSC cultures, resulting in a purity of more than 95% (Fig. 3, 4); however, as MSCs may be indirectly affected by prolonged exposure to clodronate released during macrophage death, the culture media needs to be replaced after treatment with clodrosome. Thus, the use of this method can enable the production of purified BMSCs in less than 2 weeks, thereby leading to a remarkable reduction in the time required for BMSC purification. In addition, we believe that our method will be user-friendly because it does not require careful trypsinization or frequent medium change except for the treatment with liposomal clodronate.

MSCs have the potential to differentiate into multilineage cells under certain conditions. Therefore, we needed to evaluate the functional properties of L-BMSCs generated using our method by comparing them with those of C-BMSCs. When the two types of cells were cultured in a medium that induces differentiation of cells into osteoblasts or adipocytes, the cellular differentiation as well as the expression of differentiation markers was more aggressively induced in L-BMSCs. Intriguingly, the basal levels of several differentiation marker genes, such as *Runx2*, *Sp7*, *Bsp*, *Ocn*, and *Opn* in osteoblast differentiation and *Fabp4* and *AdipoQ* in adipocyte differentiation, were significantly higher in L-BMSCs compared to C-BMSCs. In addition to the *in vitro* functional studies, the *in vivo* ectopic bone formation assay demonstrated that compared to C-BMSCs, L-BMSCs are associated with better ectopic tissue formation potential and trabecular bone structure. Collectively, we carefully assume that these differences are not the result of functional changes in BMSCs after treatment with clodrosome, but instead can be attributed to the fact that the proportion of MSCs present in the same number of cells is higher in L-BMSCs than that in C-BMSCs. These results suggest that the strategy of BMSC isolation using liposomal clodronate will be very useful in both *in vitro* and *in vivo* studies on MSCs as it would allow the generation of highly pure BMSCs with minimal macrophage contamination.

In studies employing mouse MSCs, the absence of specific markers is a disadvantage. Recently, besides contamination of hematopoietic lineage cells during mBMSC isolation, it has been demonstrated that even experimentally isolated MSCs are heterogeneous and that their cellular properties differ between individual colonies [12, 20, 25, 26]. Moreover, similar to the findings in a previous report [18, 19], we also observed that the expression of BMSC surface markers such as CD105 and CD90.2 was nonhomogeneous, as determined by immunophenotypic analysis (Fig. 4C). This observation indicates that the subpopulation of MSCs suitable for research purpose should be utilized. To address this issue, several groups have attempted to identify functionally specialized colonies by fractionating heterogeneous BMSCs based on unique MSC surface markers followed by cell sorting [12, 18, 20, 27]. Nevertheless, despite such progress, these attempts require complex procedures to separate BMs, i.e., a combination of various cell surface markers, and cell sorting systems such as fluorescence activated cell sorting (FACS) or magnetic activated cell sorting (MACS); this results in relatively low accessibility and a difficulty in establishing a standardized protocol. Furthermore, because limited MSCs exist in the mouse BM, the methods of directly sorting freshly isolated whole BM cells are likely to reduce the separation efficiency due to technical limitations [28]. Our approach allows to maximize the purity of mBMSCs (free of hematopoietic lineage cell contamination) in a short period of time. Thus, L-BMSCs could be used as source cells to improve the sorting efficiency by replacing the primary whole BM cells in studies to identify functionally differentiated MSC colonies.

There are still some obstacles to clinical application of clodrosome, although the agent has many advantages. Because effectiveness of clodrosome can be differed based on species, the effectiveness in human specimens should be confirmed. In addition, liposome can be instable to exposure of heat and irradiation in sterilization processes. Further extensive studies about the issues are needed for clinical uses.

In summary, we proposed a novel strategy for the isolation of mBMSCs, wherein liposomal clodronate was used to selectively eliminate macrophage contamination. This approach provides a standardized simple method that ensures reliable and reproducible acquisition of high-purity mBMSCs in a shorter time compared to that required upon using the conventional methods. Eventually, our protocol will help in generating source cells that have numerous applications with respect to exploring MSC biology and therapeutic potential.

## Supplementary Information

Below is the link to the electronic supplementary material.

Supplementary file1 (DOCX 39 kb)
