# Supplementary material for: Isolation of High Purity Mouse Mesenchymal Stem Cells through Depleting Macrophages Using Liposomal Clodronate
Source: Tissue Eng Regen Med. 2022 Jan 1;19(3):565–75. doi: 10.1007/s13770-021-00412-6 (PMC9130445; doi:10.1007/s13770-021-00412-6)
Supplement: Supplementary file 1 — Supplementary file1 (DOCX 39 kb) [file 13770_2021_412_MOESM1_ESM.docx]

**Supplementary Information**

**Supplementary Table S1**. List of the antibodies used in this study.

| **Target** | **Fluorophore** | **Company** | **Cat. No.** | **Isotype** |
| --- | --- | --- | --- | --- |
| CD34 | FITC | eBioscience | 11-0341-81 | Rat IgG2a, κ |
| CD45 | FITC | BioLegend | 103107 | Rat IgG2b, κ |
| CD11b | FITC | BioLegend | 101205 | Rat IgG2b, κ |
|  | Alexa 488 | BioLegend | 101219 | Rat IgG2b, κ |
|  | APC | BioLegend | 101211 | Rat IgG2b, κ |
| F4/80 | FITC | BioLegend | 123107 | Rat IgG2a, κ |
|  | PE | BioLegend | 123109 | Rat IgG2a, κ |
| Sca-1 | PE | BioLegend | 108107 | Rat IgG2a, κ |
| CD44 | PE | BioLegend | 103007 | Rat IgG2b, κ |
| CD105 | PE | eBioscience | 12-1051-81 | Rat IgG2a, κ |
| CD90.2 | PE | BioLegend | 140307 | Rat IgG2a, κ |
| Rat IgG2a, κ | FITC | BioLegend | 400505 | - |
| Rat IgG2a, κ | PE | BioLegend | 400507 | - |
| Rat IgG2b, κ | FITC | BioLegend | 400605 | - |
| Rat IgG2b, κ | PE | BioLegend | 400607 | - |
| Rat IgG2b, κ | APC | BioLegend | 400611 | - |

**Supplementary Table S2**. Primer sequences used in this study.

| **Genes** | **Sequences (5′--> 3′)** | |
| --- | --- | --- |
| *Ptprc* (Protein tyrosine phosphatase, receptor type, C; CD45) | Fw | : TTGTCACAGGGCAAACACCT |
|  | Re | : AGCGTGGATAACACACCTGG |
| *Itgam* (Integrin alpha M; CD11b) | Fw | : GAGGCCCCCAGGACTTTAAC |
|  | Re | : GCTGGACTCAGCAGGCTTTA |
| *Emr1* (EGF-like module-containing mucin-like hormone receptor-like 1; F4/80) | Fw | : CTGAACATGCAACCTGCCAC |
|  | Re | : GGTGGGACCACAGAGAGTTG |
| *CD68* (Macrosialin) | Fw | : GCTAGGACCGCTTATAGCCC |
|  | Re | : GGAGCTGGTGTGAACTGTGA |
| *Pdgfra* (platelet-derived growth factor receptor alpha) | Fw | : GGCAAAGAACAACCTCAGCG |
|  | Re | : GACTCGATAACCCTCCAGCG |
| *Runx2* (Runt-related transcription factor 2) | Fw | : TTCAACGATCTGAGATTTGTGGG |
|  | Re | : GGATGAGGAATGCGCCCTA |
| Sp7 (Transcription factor Sp7; Osterix) | Fw | : AGCGACCACTTGAGCAAACA |
|  | Re | : GCGGCTGATTGGCTTCTTCT |
| *Bsp* (Bone sialoprotein) | Fw | : AAGCAGCACCGTTGAGTATGG |
|  | Re | : CCTTGTAGTAGCTGTATTCATCCTC |
| *Ocn* (Osteocalcin) | Fw | : GCAATAAGGTAGTGAACAGACTCC |
|  | Re | : GTTTGTAGGCGGTCTTCAAGC |
| *Opn* (Osteopontin) | Fw | : GATTTGCTTTTGCCTGTTTGG |
|  | Re | : TGAGCTGCCAGAATCAGTCACT |
| *Pparg2* (Peroxisome proliferator-activated receptor gamma isoform-2) | Fw | : TCGCTGATGCACTGCCTATG |
|  | Re | : GAGAGGTCCACAGAGCTGATT |
| *Glut4* (Glucose transporter type 4) | Fw | : AATGTCCTTGCTCCAGCTCC |
|  | Re | : CAGCTCCTATGGTGGCGTAG |
| *Fabp4* (Fatty acid binding protein 4; aP2) | Fw | : CCTGGAGAAGCCGCTTATGT |
|  | Re | : AGAGTCCCGGAATGTTGCAG |
| *AdipoQ* (Adiponectin) | Fw | : CCTGGAGAAGCCGCTTATGT |
|  | Re | : AGAGTCCCGGAATGTTGCAG |
| *18s rRNA* (18S ribosomal RNA) | Fw | : GGCCGTTCTTAGTTGGTGGA |
|  | Re | : CCCGGACATCTAAGGGCATC |

Fw, forward; Re, reverse
